# Impact of family communication on self-rated health of couples who visited primary care physicians: A cross-sectional analysis of Family Cohort Study in Primary Care

**DOI:** 10.1371/journal.pone.0213427

**Published:** 2019-03-13

**Authors:** Seo Young Kang, Jung Ah Lee, Young Sik Kim

**Affiliations:** Department of Family Medicine, Asan Medical Center, University of Ulsan College of Medicine, Seoul, Korea; University of Colorado Denver, UNITED STATES

## Abstract

**Introduction:**

Self-rated health (SRH) is a subjective health measurement that predicts mortality and morbidity and reflects mental health and socioeconomic status. Since a couple’s relationship can influence the health status of the individuals involved, poor family communication can negatively influence the health status of its members. The aim of this study was to investigate the factors affecting SRH among married couples in primary care and evaluated the effect of family communication on SRH.

**Material and methods:**

In this cross-sectional analysis of Family Cohort Study in Primary Care, 469 couples (938 participants) were analyzed to evaluate the relationship between SRH and family communication. Participants answered questionnaires on demographic characteristics and lifestyle factors. The Korean version of the Family Communication Scale of Family Adaptation and Cohesion Evaluation Scale-IV was used to assess family communication, and a 5-point scale of SRH questions was used to assess the SRH status. Multivariate logistic regression analyses were performed in order to evaluate the relationship between family communication and SRH and identify associated factors for good SRH.

**Results:**

Wives with a high family communication level had higher OR for good SRH. When the husband and wife both reported high family communication levels, the OR for good SRH increased in wives; however, the relationship between family communication and SRH was not significant in husbands. In the multi-adjusted model, the OR for good SRH of husbands increased in those with >12 years of education, moderate drinkers and decreased in current smokers. The OR for good SRH of wives increased in those with age of 60 to 69, those with >12 years of education, and those who participated in vigorous physical activity, and decreased in those with diabetes and depressive mood.

**Conclusions:**

Our results indicate that improvement in family communication may contribute to better SRH.

## Introduction

Self-rated health (SRH) is a subjective health measurement, which can be easily assessed using a single question rating one’s current health status. A number of studies have consistently reported that SRH is a good predicting factor of mortality and morbidity [1–4]. People with poor SRH were more frequently hospitalized and utilized health care [4]. Furthermore, SRH is associated with life satisfaction and mental health status as well as socioeconomic status [5, 6]. Although the mechanism of using SRH as a representation of general health assessment is poorly understood, several studies identified SRH as a valid measure of general health in many occasions [7–9].

The relationship between married couples or family members can influence the health status of the individuals involved. Furthermore, the dynamics within a family influence SRH of individuals because families are an essential part of one’s general health. For instance, marital termination, such as divorce or death of a spouse can negatively impact the health status of a person directly involved in these situations [10]. Stressful events in a relationship can also negatively influence one’s health status. In elderly couples, serious spousal illness or hospitalization aggravated the health status of their partners and led to low SRH [11].

The family communication scale (FCS) evaluates listening skills, speaking skills, self-disclosure, clarity, continuity tracking, and respect and regard of the family [12]. The FCS is one of the three dimensions in the Circumplex Model, which was initially developed in order to assess family communications between adolescents and their parents; however, it can also evaluate levels of communication within couples [12, 13]. Family communication facilitates the movement of the two dimensions of family dynamics; adaptability and cohesion [12]. Therefore, positive communication between couples increases awareness of current needs and improves problem-solving skills [14]. Conversely, negative communication between couples can increase health risk behaviors such as problematic drinking [15].

Although a previous study reported the association between spousal illness and SRH [11], very few studies have evaluated the relations between family dynamics and SRH. Additionally, SRH and its associated factors were not thoroughly evaluated among couples, who seek primary care for chronic diseases. In this study, we aimed to evaluate the association between family communication and SRH in husbands and wives and investigated the associated factors of SRH among married couples in primary care.

## Materials and methods

### Study participants

This cross-sectional study was based on data collected between April 2009 and June 2011 for the FACTS (Family Cohort Study in Primary Care). For this cohort, 28 family physicians in 22 hospitals in Korea recruited married couples, who visited hospital-based family practices for treatment of chronic diseases or general health examinations.

There was no prescreening of the participants; therefore, physicians asked all married couples, who came to the outpatient clinics, whether to register in this cohort. If couples agreed and signed the informed consent, physicians collected data using standardized questionnaires. Patients who had difficulty understanding and writing responses to the questionnaire were excluded. In the end, 520 couples (1,040 participants) were enrolled. The study protocol and the written informed consent form were approved by the Institutional Review Board of Asan Medical Center (IRB number 2009–0347). All participants read and signed the informed consent form before participating in the study.

Among the 1,040 study participants, we excluded 5 participants, who did not provide answers to the SRH questionnaires, 46 participants who did not provide answers to the Korean version of the FCS of the FACE-IV (Family Adaptation and Cohesion Evaluation Scale), and 8 participants who neglected to provide answers for both the SRH and FCS questionnaires. Additionally, we excluded 43 participants whose spouses were excluded for not completing the questionnaires. In total, 51 couples (102 participants) were excluded, and 469 couples (938 participants) were analyzed to investigate the relationship between SRH and family communication.

### Data collection and measurement

All of the physicians collected data using standardized questionnaires that included questions regarding demographic characteristics, such as age, sex, educational level, and income, as well as lifestyle factors, such as smoking habits, alcohol consumption, and physical activity. The participants answered the questionnaires individually, which were then reviewed by trained interviewers or their physicians. Educational level was categorized into three groups: <12 years, 12 years and >12 years, and monthly household income was divided into four groups: <2 million won (1,761 USD), 2–4 million won (1,761–3,521 USD), 4–6 million won (3,521–5,282 USD), and ≥6 million won (5,282 USD). Smoking status was determined as nonsmoker, ex-smoker, and current smoker, and alcohol consumption was categorized as non-drinker, moderate drinker, and heavy drinker according to the National Institute on Alcohol Abuse and Alcoholism (NIAAA) definitions. Heavy drinkers were defined as men who drank more than 14 glasses per week and women who drank more than 7 glasses per week [16]. Physical activity was measured using the International Physical Activity Questionnaire Short-Form (IPAQ-SF), which is a scale based on activity intensity and exercise time during the previous 7 days [17]. In addition, information about previous medical history and concomitant medications was collected. Participants also answered Center for Epidemiologic Studies Depression Scale (CES-D) questionnaires, and a depressive mood was defined when participants were diagnosed with depression or had a CES-D score ≥ 21 [18].

### Measurement of family functioning and communication

Each individual answered questionnaires for family functioning and family communication, which were then evaluated independently regardless of one’s spouse’s answer. Family functioning was evaluated using a Korean version of the FACES-III. Developed by Olson and colleagues to measure the quality of family relations, FACES-III consists of 10 questions assessing family adaptability and 10 questions assessing family cohesion [19]. Family functioning is defined by the combination of the categories of family adaptability and cohesion and is classified into balanced, mid-range, and extreme family types.

Family communication was evaluated using the Korean version of the FCS of the FACES-IV [20]. The FCS consists of 10 questions scored on a scale ranging from 1 to 5. According to the sum of the scores, family communication is classified into low (10–35), moderate (36–39), and high (40–50) groups. Higher scores represent better family communication (S1 Appendix).

### Measurement of SRH

We asked the following question to assess the SRH status of the participants: “In general, how would you rate your health?” Patients responded using a 5-point scale of excellent, very good, good, fair, and poor [21]. Excellent, very good, and good SRH status were categorized as good SRH status, while fair and poor SRH status were grouped as poor SRH status.

### Statistical analyses

The Pearson’s chi-square test and Fisher’s exact test were used to compare the characteristics of the patients with good SRH status and poor SRH status. Logistic regression models were used to estimate the odds ratios (ORs) and 95% confidence intervals (CIs) of good SRH status in association with each level of family communication. Furthermore, ORs and 95% CIs of the variables associated with good SRH were obtained. We adjusted for age and all covariates that were found to be significant from the Pearson’s chi-square test, and analyses were separately performed for husbands and wives. For sensitivity analyses, the analyses were also performed in combination of both sexes. Lastly, we obtained ORs and 95% CIs of good SRH status according to spousal combinations of family communication levels. Further sensitivity analyses were performed after stratifying according to the educational level. We also investigated the relationship between family communication and several health conditions by logistic regression analyses. In this study, p < 0.05 was considered statistically significant, and all data were analyzed using IBM SPSS Statistics for Windows, Version 23.0 (IBM Corp., Armonk, NY, USA).

## Results

### Basic characteristics of the study participants

Table 1 lists the basic characteristics of the study participants. All participants included in the analysis were married couples. The mean age (standard deviation) was 57.35(10.12) years, and most participants (68.1%) were between the ages of 50 and 69. Among the participants, 48.7% had >12 years of education, indicating that approximately half of them finished high school. The monthly household income was ≥6 million won (5,282 USD) for 29.3% of the participants. Of the 12.3% of participants who were current smokers, the majority were men (21.9% of husbands and 1.5% of wives). Similarly, most of the heavy drinkers (16.7% of participants) were men (27.9% of husbands and 5.4% of wives). Vigorous physical activity was observed in 36.1% of the participants. Diabetes was present in 20.8%, while hypertension and dyslipidemia were present in 41.5% and 36.7% of the participants, respectively. Depressive mood was observed in 15.2% of the participants.

**Table 1:** Basic characteristics of the 938 study participants.

	Total (n = 938)		Husband (n = 469)		Wife (n = 469)	
	N	%	N	%	N	%
^a^Age (years)	57.35	10.12	59.07	10.17	55.62	9.77
Education (years)						
<12	182	19.4	63	13.4	119	25.4
12	298	31.8	128	27.3	170	36.2
>12	455	48.5	277	59.1	178	38.0
Missing	3	0.3	1	0.2	2	0.4
Household income (10,000won/month)						
<200 (1,761 USD)	164	17.5	79	16.8	85	18.1
200–399 (1,761–3,521 USD)	279	29.7	141	30.1	138	29.4
400–599 (3,521–5,282 USD)	202	21.5	101	21.5	101	21.5
≥600 (5,282 USD)	267	28.5	141	30.1	126	26.9
Missing	26	2.8	7	1.5	19	4.1
Smoking status						
Nonsmoker	465	49.6	80	17.1	385	82.1
Ex-smoker	279	29.7	269	57.4	10	2.1
Current smoker	104	11.1	98	20.9	6	1.3
Missing	90	9.6	22	4.7	68	14.5
Alcohol consumption						
Non-drinker	376	40.1	118	25.2	258	55.0
Moderate drinker	393	41.9	216	46.1	177	37.7
Heavy drinker	154	16.4	129	27.5	25	5.3
Missing	15	1.6	6	1.3	9	1.9
Physical activity						
Inactive	242	25.8	114	24.3	128	27.3
Minimally active	295	31.4	148	31.6	147	31.3
Vigorous	304	32.4	176	37.5	128	27.3
Missing	97	10.3	31	6.6	66	14.1
Diseases or conditions						
Hypertension	379	41.5	216	47.3	163	35.7
Dyslipidemia	335	36.7	183	40.0	152	33.3
Diabetes Mellitus	190	20.8	129	28.2	61	13.4
Depressive mood	132	15.2	46	10.6	86	19.8
Family functioning						
Balanced	210	22.4	105	22.4	105	22.4
Midrange	408	43.5	209	44.6	199	42.4
Extreme	183	19.5	95	20.3	88	18.8
Missing	137	14.6	60	12.8	77	16.4
Family communication						
Low	206	22.0	106	22.6	100	21.3
Moderate	259	27.6	123	26.2	136	29.0
High	473	50.4	240	51.2	233	49.7
Self-rated health status						
Excellent	30	3.2	17	3.6	13	2.8
Very good	162	17.3	109	23.2	53	11.3
Good	269	28.7	153	32.6	116	24.7
Fair	366	39.0	151	32.2	215	45.8
Poor	111	11.8	39	8.3	72	15.4

^a^Mean and standard deviation are presented.

A balanced family type was observed in 26.2% of the participants. Family communication levels were low in 22.0%, moderate in 27.6%, and high in 50.4% of the participants. When assessing one’s health status, 49.2% reported his or her health status to be excellent, very good, or good, while 50.8% reported fair or poor health status.

### Factors associated with good SRH

Table 2 shows the distribution of SRH status of husbands and wives according to demographic, lifestyle factors and family dynamics. The frequency of good SRH status of husbands was higher in those with higher educational levels (68.1% for >12 years of education) and incomes (33.0% for ≥6 million won [5,282 USD]) nonsmokers (21.4%) and ex-smokers (60.5%) (p < 0.05). Although family functioning was not significantly different between the two groups (p = 0.660), husbands with higher family communication levels were more likely to be in a good SRH status (p = 0.034). Furthermore, husbands with depressive mood were more likely to be in a poor SRH status (p = 0.006). The frequency of good SRH status of wives was higher in those with higher educational levels (50.8% for >12 years of education) and incomes (35.5% for ≥6 million won [5,282 USD]). Wives with higher family communication levels were more likely to be in a good SRH status, while wives with diabetes, hypertension, and depressive mood were more likely to be in a poor SRH status (p < 0.05).

**Table 2:** Distribution of SRH status of husbands and wives.

	Husbands			Wives		
	Good SRH (n = 279)	Poor SRH (n = 190)	p-value^a^	Good SRH (n = 182)	Poor SRH (n = 287)	p-value^a^
Age (years)						
<50	60 (21.5)	27 (14.2)	0.684	48 (26.4)	67 (23.3)	0.979
50–59	66 (23.7)	63 (33.2)		61 (33.5)	115 (40.1)	
60–69	108 (38.7)	72 (37.9)		64 (35.2)	90 (31.4)	
≥ 70	45 (16.1)	28 (14.7)		9 (4.9)	15 (5.2)	
Education (years)						
<12	25 (9.0)	38 (20.1)		33 (18.2)	86 (30.1)	
12	64 (22.9)	64 (33.9)	< 0.001*	56 (30.9)	114 (39.9)	< 0.001*
>12	190 (68.1)	87 (46.0)		92 (50.8)	86 (30.1)	
Household income (10,000won/month)						
<200 (1,761 USD)	37 (13.4)	42 (22.6)	0.010*	23 (13.4)	62 (22.3)	< 0.001*
200–399 (1,761–3,521 USD)	82 (29.7)	59 (31.7)		41 (23.8)	97 (34.9)	
400–599 (3,521–5,282 USD)	66 (23.9)	35 (18.8)		47 (27.3)	54 (19.4)	
≥600 (5,282 USD)	91 (33.0)	50 (26.9)		61 (35.5)	65 (23.4)	
Smoking status						
Nonsmoker	57 (21.4)	23 (12.7)		150 (96.2)	235 (95.9)	
Ex-smoker	161 (60.5)	108 (59.7)	0.003*	6 (3.8)	4 (1.6)	0.360
Current smoker	48 (18.0)	50 (27.6)		0 (0.0)	6 (2.4)	
Alcohol consumption						
Non-drinker	55 (19.9)	63 (33.7)		97 (54.8)	161 (56.9)	
Moderate drinker	145 (52.5)	71 (38.0)	0.061	72 (40.7)	105 (37.1)	0.917
Heavy drinker	76 (27.5)	53 (28.3)		8 (4.5)	17 (6.0)	
Physical activity						
Inactive	61 (22.9)	53 (30.8)		45 (28.7)	83 (33.7)	
Minimally active	91 (34.2)	57 (33.1)	0.061	56 (35.7)	91 (37.0)	0.159
Vigorous	114 (42.9)	62 (36.0)		56 (35.7)	72 (29.3)	
Diseases or conditions						
Hypertension	120 (43.8)	96 (52.5)	0.070	52 (29.1)	111 (39.9)	0.021*
Dyslipidemia	105 (38.3)	78 (42.6)	0.381	56 (31.3)	96 (34.5)	0.479
Diabetes Mellitus	68 (24.8)	61 (33.3)	0.056	13 (7.3)	48 (17.3)	0.002*
Depressive mood	19 (7.2)	27 (15.7)	0.006*	19 (10.9)	67 (25.8)	< 0.001*
Family functioning						
Balanced	59 (24.3)	46 (27.7)		46 (29.3)	59 (25.1)	
Midrange	134 (55.1)	75 (45.2)	0.660	75 (47.8)	124 (52.8)	0.639
Extreme	50 (20.6)	45 (27.1)		36 (22.9)	52 (22.1)	
Family communication						
Low	54 (19.4)	52 (27.4)		30 (16.5)	70 (24.4)	
Moderate	73 (26.2)	50 (26.3)	0.034*	46 (25.3)	90 (31.4)	0.004*
High	152 (54.5)	88 (46.3)		106 (58.2)	127 (44.3)	

^a^P for trends are presented for (2 x n) data using linear by linear association

*P < 0.05

The ORs for good SRH according to potential risk factors are presented in Table 3. In the multi-adjusted model of husbands, the OR for good SRH significantly increased in those with >12 years of education (OR 3.80, 95% CI 1.87–7.73), moderate drinkers (OR 2.47, 95% CI 1.43–4.29) and decreased in current smokers (OR 0.44, 95% CI 0.22–0.89) after adjusting for age, educational level, income, smoking status, depressive mood, and family communication level. In the multi-adjusted model of wives, the OR for good SRH significantly increased in those with age of 60 to 69 (OR 2.10, 95% CI 1.10–3.99), those with >12 years of education (OR 2.48, 95% CI 1.32–4.65), and those who participated in vigorous physical activity (OR 1.89, 95% CI 1.03–3.47) after adjusting for age, educational level, income, hypertension, diabetes, depressive mood, and family communication level. The OR for good SRH decreased in wives with diabetes (OR 0.29, 95% CI 0.14–0.62) and depressive mood (OR 0.45, 95% CI 0.24–0.86). The results were similar when both sexes were analyzed together. The OR for good SRH increased in those with >12 years of education (OR 3.32, 95% CI 1.94–5.68), moderate drinkers (OR 1.83, 95% CI 1.25–2.69), and those who participated in vigorous physical activity (OR 1.67, 95% CI 1.10–2.55) and decreased in those with diabetes (OR 0.63, 95% CI 0.41–0.98) and depressive mood (OR 0.48, 95% CI 0.28–0.83). The OR for good SRH was lower in wives (OR 0.34, 95% CI 0.19–0.61) compared with husbands (S1 Table).

**Table 3:** The ORs for good SRH according to potential risk factors in husbands and wives.

	Husbands (n = 469)				Wives (n = 469)			
	Crude		Multi-adjusted^a^		Crude		Multi-adjusted^b^	
	OR	95% CI	OR	95% CI	OR	95% CI	OR	95% CI
Age (years)								
<50	1.00	-	1.00	-	1.00	-	1.00	-
50–59	0.47*	0.27–0.83	0.56	0.29–1.08	0.74	0.46–1.20	1.14	0.63–2.04
60–69	0.68	0.39–1.16	0.86	0.45–1.62	0.99	0.61–1.62	2.10*	1.10–3.99
≥ 70	0.72	0.38–1.39	0.79	0.36–1.73	0.84	0.34–2.07	2.91	0.94–8.99
Education (years)								
<12	1.00	-	1.00	-	1.00	-	1.00	-
12	1.52	0.82–2.80	1.69	0.82–3.48	1.28	0.77–2.14	1.20	0.65–2.24
>12	3.32*	1.89–5.84	3.80*	1.87–7.73	2.79*	1.70–4.59	2.48*	1.32–4.65
Household income (10,000won/month)								
<200 (1,761 USD)	1.00	-	1.00	-	1.00	-	1.00	-
200–399 (1,761–3,521 USD)	1.58	0.91–2.75	1.08	0.54–2.17	1.14	0.62–2.08	0.89	0.45–1.79
400–599 (3,521–5,282 USD)	2.14*	1.17–3.91	1.22	0.57–2.60	2.35*	1.27–4.35	1.62	0.77–3.39
≥600 (5,282 USD)	2.07*	1.18–3.62	1.02	0.50–2.11	2.53*	1.40–4.58	1.56	0.77–3.17
Smoking status								
Nonsmoker	1.00	-	1.00	-	1.00	-	1.00	-
Ex-smoker	0.60	0.35–1.03	0.59	0.32–1.07	2.35	0.65–8.47	3.71	0.82–16.83
Current smoker	0.39*	0.21–0.72	0.44*	0.22–0.89	NA	NA	NA	NA
Alcohol consumption								
Non-drinker	1.00	-	1.00	-	1.00	-	1.00	-
Moderate drinker	2.34*	1.48–3.71	2.47*	1.43–4.29	1.14	0.77–1.68	1.30	0.81–2.08
Heavy drinker	1.64	0.99–2.72	1.68	0.89–3.17	0.78	0.33–1.88	0.80	0.29–2.25
Physical activity								
Inactive	1.00	-	1.00	-	1.00	-	1.00	-
Minimally active	1.39	0.85–2.28	1.06	0.60–1.87	1.14	0.69–1.86	1.21	0.68–2.17
Vigorous	1.60	0.99–2.58	1.41	0.81–2.45	1.44	0.87–2.37	1.89*	1.03–3.47
Diseases or conditions								
Hypertension	0.71	0.49–1.03	0.69	0.44–1.09	0.62*	0.41–0.92	0.69	0.42–1.15
Dyslipidemia	0.84	0.57–1.22	0.65	0.38–1.11	0.86	0.58–1.29	0.91	0.55–1.50
Diabetes Mellitus	0.66*	0.44–0.99	0.86	0.53–1.39	0.37*	0.20–0.71	0.29*	0.14–0.62
Depressive mood	0.42*	0.23–0.78	0.49	0.24–1.01	0.35*	0.20–0.61	0.45*	0.24–0.86
Family functioning								
Balanced	1.00	-	1.00	-	1.00	-	1.00	-
Midrange	1.39	0.86–2.25	1.60	0.91–2.82	0.78	0.48–1.25	1.63	0.95–2.79
Extreme	0.87	0.50–1.51	0.99	0.51–1.94	0.89	0.50–1.58	0.98	0.51–1.87
Family communication								
Low	1.00	-	1.00	-	1.00	-	1.00	-
Moderate	1.41	0.83–2.37	1.60	0.87–2.94	1.19	0.68–2.08	1.11	0.58–2.11
High	1.66*	1.05–2.64	1.66	0.97–2.85	1.95*	1.18–3.21	1.91*	1.07–3.41

^a^Adjusted for age, educational level, income, smoking status, depressive mood, and family communication level

^b^Adjusted for age, educational level, income, hypertension, diabetes, depressive mood, and family communication level

*P < 0.05

### Association between family communication and SRH

The OR for good SRH increased in wives with high family communication levels (OR 1.91, 95% CI 1.07–3.41); however, the relationship was not significant in husbands (Table 3). This finding was consistent in participants with > 12 years of education (S2 Table). However, there was no significant relationship between family communication and SRH when participants had ≤ 12 years of education. When both sexes were analyzed together, the OR for good SRH increased in those with high family communication levels (OR 2.07, 95% CI 1.33–3.23) (S1 Table). Table 4 presents the ORs for good SRH status according to spousal combinations of family communication levels. The OR for good SRH of wives significantly increased when the husband and wife both reported high family communication levels (OR 2.31, 95% CI 1.32–4.05); however, the relationship was not significant in husbands. This finding was consistent when participants were stratified according to educational level (S3 Table).

**Table 4:** Relationship between good SRH status and spousal combinations of family communication levels.

Family communication levels (Husband + Wife) (n = 938)	Crude		Multi-adjusted^a^	
	OR	95% CI	OR	95% CI
Husbands				
Moderate/Low + Moderate/Low	1.00	-	1.00	-
Moderate/Low + High	0.85	0.47–1.53	0.94	0.48–1.86
High + Moderate/Low	0.86	0.49–1.52	1.07	0.55–2.09
High + High	1.59*	1.02–2.48	1.31	0.78–2.19
Wives				
Moderate/Low + Moderate/Low	1.00	-	1.00	-
Moderate/Low + High	1.45	0.79–2.64	1.86	0.87–3.99
High + Moderate/Low	0.76	0.41–1.40	1.48	0.65–3.38
High + High	1.69*	1.09–2.62	2.31*	1.32–4.05

^a^Adjusted for age, educational level, income, smoking status, hypertension, diabetes, and depressive mood

*P < 0.05

S4 Table presents the ORs for health conditions according to family communication levels. The OR for depressive mood increased as family communication aggravated in both husbands and wives. The associations between family communication and hypertension, diabetes mellitus, and dyslipidemia were not significant.

## Discussion

In this study, we identified the associated factors of SRH among married couples and examined the relationship between family communication on SRH among husbands and wives for the first time. In the analyses of husbands, we found that men with high educational level and men who drank moderately reported good SRH, and current smokers were more likely to have poor SRH. In the analyses of wives, women in their sixties, women with high educational level, and women who participate in vigorous physical activity reported good SRH, while women with diabetes and depressive mood were more likely to be in a poor SRH status. Furthermore, wives with a high level of communication had good SRH, although family communication did not influence husbands’ SRH. Additionally, wives were more likely to report good SRH when the husband and the wife both reported high communication levels.

SRH is a strong predictor of mortality in both acute and chronic contexts [1, 3]. It also influences the incidence of chronic diseases such as coronary heart disease, diabetes, stroke, lung disease, and arthritis [2]. In our crude analyses, husbands with diabetes and depressive mood had poor SRH, while wives with diabetes, hypertension, and depressive mood reported poor SRH. However, after adjusting for potential risk factors, only the association with diabetes mellitus and depressive mood remained significant for wives. Unlike patients with hypertension or dyslipidemia, patients with diabetes have to undertake several self-care activities in order to avoid microvascular and macrovascular complications of diabetes. They need to monitor blood glucose levels, take medications regularly, inject insulins, eat healthy food, and participate in regular physical activity. These activities may cause social stigma to diabetic patients and make them to assess their health status as poor [22]. Wives with depressive mood according to depression diagnosis or CES-D score ≥21 also demonstrated poor SRH. Since CES-D is a self-reporting questionnaire, those who reported themselves as depressed would also be likely to assess their health status as poor [18]. The relationship between comorbidities and SRH was significant only in wives, which implies that women are psychologically more vulnerable to medical conditions and recognize their medical conditions negatively. It has been reported that male diabetics experience lesser depression and anxiety but more energy and better positive wellbeing [23].

When the relationship between family communication and health conditions was analyzed, depressive mood was the only comorbidity associated with family communication. Emotional regulation is the essential aspect of the etiology of depression, and family communication is critical in facilitating emotional understanding [24]. Therefore, poor family communication may contribute to depressive symptoms. On the other hand, depressive symptoms can also aggravate family dynamics by influencing the mood of the family members [25]. Although the cross-sectional nature of our analysis does not demonstrate a definite direction between family communication and depressive mood, it certainly illustrates the role of psychological health on family communication and vice versa.

Among the lifestyle factors, moderate drinking and vigorous physical activity were associated with good SRH, and current smoking was associated with poor SRH. Men who currently smoke were more likely to be in a poor SRH status, while men who drink moderately reported good SRH. Women, who participate in vigorous physical activity, had good SRH. Harmful effects of cigarette smoking are well recognized nowadays due to various anti-smoking campaigns and actions targeted for general population; thus, current smokers were likely to report their health status as poor. The analysis for women was inconclusive because the number of ex-smokers and current smokers were very few among the wives.

Since the study design is cross-sectional, participants with good health status or who were confident in their own health were likely to be moderate drinkers. This phenomenon was also observed in a previous study that reported an association between good SRH and occasional and moderate drinking [26]. Unlike the study of Valencia-Martin, which included both genders, the relationship between moderate drinking and good SRH was significant only for men in our analysis. This may imply that women, who are confident in their own health, do not engage in drinking behavior as much as healthy men do.

Physical activity is beneficial for both physical fitness and mental health [27]. Previous studies have shown that an increase in moderate-to-vigorous physical activity was associated with improvement in health-related quality of life, and increased physical activity also improved quality of life in patients with comorbidities [28, 29]. Likewise, in our study, vigorous physical activity was associated with good SRH after adjusting for comorbidities such as diabetes and hypertension. The relationship between vigorous physical activity and good SRH was observed only for women in our analysis. In a previous study by Morimoto et al, the amount of physical activity had positive effects on quality of life for both men and women; however, women had more preferable effects of maximum intensity on quality of life than men [30]. Further investigation is needed to interpret the gender differences of physical activity on SRH.

While high educational level was significantly associated with good SRH, we did not find a significant association between income and SRH. Although socioeconomic status can be expressed in terms of income or occupation, we might also suggest that one’s level of education reflects his or her fundamental social position because childhood education depends mostly on one’s parents’ socioeconomic status, and education determines an individual’s occupation and income [31]. Educational attainment was previously reported as a predictor of life expectancy, and people with lower educational level had poor SRH and shorter life expectancy in comparison to the more educated [31, 32].

In our analyses, wives in their sixties had approximately twice higher odds of good SRH in comparison to the wives whose age is less than 50. In general, the proportions of good SRH decrease according to age since both physical and mental health conditions decline with aging [33]. One possible explanation of increased good SRH in sixties in our study would be that Korean women in sixties often become free from domestic work related to children’s education and from housework because this period is when their husbands retire. It has been reported that housework may increase stress and decrease perceived well-being of individuals [34, 35]. Age-specific distribution of SRH of a larger Korean population would be an interest of future research.

Wives with a high level of family communication demonstrated good SRH, which implies that communication between couples is an important determinant of health, especially for women. When SRH status was analyzed according to spousal combinations of family communication levels, good SRH significantly increased in wives when the husband and the wife both reported high family communication levels. However, the relationship between SRH and family communication was not significant among the husbands. Family communication has a significant association with a variety of cognitive activities and relational behaviors, as well as individual well-being [36]. Therefore, high family communication facilitates interaction between family members and can positively influence the mental status of family members. Conversely, low family communication can have negative impact on psychological status of family members, which make individuals to report poor SRH. In addition, family communication can be deteriorated when spouses are not healthy. For instance, poor family communication was reported in spouses of patients with Parkinsonism [37]. In our analyses, the relationship between SRH and family communication was observed only in wives. This suggests that psychological aspects of women are more likely to be influenced by environmental factors in comparison to those of men as reported in a previous study [38]. In the subgroup analyses stratified by educational level, the association between family communication and SRH remained significant only in wives with > 12 years of education; whereas, the association between SRH and spousal combinations of family communication levels was significant in wives regardless of their educational level. This finding suggests the possible influence of education on the association between family communication and SRH.

In our study, we evaluated SRH as the only measure of general health. Previous studies have identified SRH as a valid measure of general health although the mechanism is not well understood [7–9]. It predicts mortality, morbidity, and even mental health [1–5]. There are social and biological pathways that mediate certain health information from the human body to consciousness, which contribute self-rating of health [7]. We evaluated husbands and wives separately because questionnaires were completed separately although they were couples.

Our study has a few limitations. Since the study design is cross-sectional, we cannot determine whether a high communication level causes good SRH or vice versa. In addition, the study data were obtained with self-reporting questionnaires; thus, the responses could be exaggerated or downscaled. Conversely, this is meaningful in a way that SRH evaluates subjective health status and reflects the quality of life of each participant. We only used SRH as the measure of health; therefore, unmeasured heterogeneity may exist in our analyses. Furthermore, there is a selection bias regarding the following three points. First, the study participants were limited to patients receiving primary care; therefore, our results might not be applicable to the general healthy population. Second, participants who agreed to enroll in the cohort may have different characteristics from those who did not agree to enroll in the cohort. Third, the number of patients varies among the 28 family physicians. However, to the best of our knowledge, this is the first study to investigate the association between family communication and SRH in married couples who sought primary care. We only evaluated family communication between spouses and our analyses did not include family communication between other family members although FCS was initially developed for assessing family communication between parents and children [12]. However, FCS had been used to assess family communication between spouses in previous studies [13]. In this study, we analyzed the relationship between SRH and family communication in several ways, including on an individual basis as well as in terms of spousal combinations. Furthermore, we evaluated gender-specific factors that were associated with good SRH of husbands and wives.

## Conclusions

In this study, wives with a high level of family communication had good SRH, and they were more likely to report good SRH when the husband and the wife both reported high communication levels. However, family communication was not associated with husbands’ SRH. Additionally, men with high educational level and men who drank moderately reported good SRH, and men who currently smoke were more likely to have poor SRH. Women in their sixties, women with high educational level, and women who participate in vigorous physical activity reported good SRH, while women with diabetes and depressive mood were more likely to be in a poor SRH status. Our results indicate that improvement in family communication may contribute to better SRH.

## Supporting information

S1 Dataset Data for analyses. (SAV)

S1 Table The ORs for good SRH according to potential risk factors in both sexes. (DOCX)

S2 Table Relationship between good SRH and family communication according to educational level. (DOCX)

S3 Table Relationship between good SRH status and spousal combinations of family communication levels according to educational level. (DOCX)

S4 Table Relationship between family communication and health conditions. (DOCX)

S1 Appendix The Family Communication Scale of the Family Adaptation and Cohesion Evaluation Scale–IV (English version). (DOCX)
