# Supplementary material for: Impact of family communication on self-rated health of couples who visited primary care physicians: A cross-sectional analysis of Family Cohort Study in Primary Care
Source: PLoS One. 2019 Mar 13;14(3):e0213427. doi: 10.1371/journal.pone.0213427 (PMC6415836; doi:10.1371/journal.pone.0213427)
Supplement: S1 Table — (DOCX) [file pone.0213427.s002.docx]

**S1 Table. The ORs for good SRH according to potential risk factors in both sexes**

|  | Crude | | Multi-adjusted^a^ | |
| --- | --- | --- | --- | --- |
|  | OR | 95% CI | OR | 95% CI |
| Age (years) |  |  |  |  |
| <50 | 1.00 | - | 1.00 | - |
| 50-59 | 0.62^*^ | 0.43-0.89 | 0.82 | 0.51-1.32 |
| 60-69 | 0.92 | 0.65-1.31 | 1.40 | 0.84-2.33 |
| ≥ 70 | 1.09 | 0.67-1.78 | 1.44 | 0.71-2.92 |
| Sex |  |  |  |  |
| Male | 1.00 | - | 1.00 | - |
| Female | 0.43^*^ | 0.33-0.56 | 0.34^*^ | 0.19-0.61 |
| Education (years) |  |  |  |  |
| <12 | 1.00 | - | 1.00 | - |
| 12 | 1.44 | 0.98-2.13 | 1.40 | 0.82-2.41 |
| >12 | 3.49^*^ | 2.42-5.02 | 3.32^*^ | 1.94-5.68 |
| Household income (10,000won/month) |  |  |  |  |
| <200 (1,761 USD) | 1.00 | - | 1.00 | - |
| 200-399 (1,761-3,521 USD) | 1.37 | 0.92-2.03 | 1.07 | 0.62-1.86 |
| 400-599 (3,521-5,282 USD) | 2.20^*^ | 1.44-3.36 | 1.38 | 0.77-2.50 |
| ≥600 (5,282 USD) | 2.29^*^ | 1.54-3.42 | 1.43 | 0.81-2.53 |
| Smoking status |  |  |  |  |
| Nonsmoker | 1.00 | - | 1.00 | - |
| Ex-smoker | 1.86^*^ | 1.38-2.51 | 0.66 | 0.37-1.18 |
| Current smoker | 1.07 | 0.70-1.64 | 0.59 | 0.29-1.18 |
| Alcohol consumption |  |  |  |  |
| Non-drinker | 1.00 | - | 1.00 | - |
| Moderate drinker | 1.82^*^ | 1.37-2.42 | 1.83^*^ | 1.25-2.69 |
| Heavy drinker | 1.77^*^ | 1.21-2.58 | 1.13 | 0.66-1.93 |
| Physical activity |  |  |  |  |
| Inactive | 1.00 | - | 1.00 | - |
| Minimally active | 1.27 | 0.91-1.79 | 1.18 | 0.78-1.80 |
| Vigorous | 1.63^*^ | 1.16-2.29 | 1.67^*^ | 1.10-2.55 |
| Diseases or conditions |  |  |  |  |
| Diabetes Mellitus | 0.70^*^ | 0.51-0.97 | 0.63^*^ | 0.41-0.98 |
| Hypertension | 0.75^*^ | 0.58-0.98 | 0.77 | 0.52-1.12 |
| Dyslipidemia | 0.91 | 0.70-1.19 | 0.73 | 0.50-1.07 |
| Depressive mood | 0.34^*^ | 0.23-0.51 | 0.48^*^ | 0.28-0.83 |
| Family functioning |  |  |  |  |
| Balanced | 1.00 | - | 1.00 | - |
| Midrange | 1.05 | 0.75-1.47 | 1.35 | 0.87-2.10 |
| Extreme | 0.89 | 0.60-1.32 | 1.02 | 0.60-1.75 |
| Family communication |  |  |  |  |
| Low | 1.00 | - | 1.00 | - |
| Moderate | 1.24 | 0.85-1.79 | 1.39 | 0.85-2.25 |
| High | 1.74^*^ | 1.25-2.43 | 2.07^*^ | 1.33-3.23 |

^a^Adjusted for age, sex, educational level, income, smoking status, alcohol consumption, physical activity, hypertension, diabetes mellitus, depressive mood, and family communication level

^*^P < 0.05
