# Supplementary material for: Impact of family communication on self-rated health of couples who visited primary care physicians: A cross-sectional analysis of Family Cohort Study in Primary Care
Source: PLoS One. 2019 Mar 13;14(3):e0213427. doi: 10.1371/journal.pone.0213427 (PMC6415836; doi:10.1371/journal.pone.0213427)
Supplement: S2 Table — (DOCX) [file pone.0213427.s003.docx]

**S2 Table. Relationship between good SRH and family communication according to educational level.**

| **Educational level** | Husbands | | | | Wives | | | |
| --- | --- | --- | --- | --- | --- | --- | --- | --- |
|  | Crude | | Multi-adjusted^a^ | | Crude | | Multi-adjusted^b^ | |
|  | OR | 95% CI | OR | 95% CI | OR | 95% CI | OR | 95% CI |
| **≤12 years** |  |  |  |  |  |  |  |  |
| Family communication |  |  |  |  |  |  |  |  |
| Low | 1.00 | - | 1.00 | - | 1.00 | - | 1.00 | - |
| Moderate | 1.27 | 0.55-2.92 | 2.06 | 0.78-5.45 | 0.97 | 0.45-2.08 | 0.89 | 0.37-2.16 |
| High | 1.39 | 0.68-2.84 | 1.45 | 0.61-3.49 | 1.77 | 0.90-3.47 | 1.73 | 0.79-3.79 |
| **>12 years** |  |  |  |  |  |  |  |  |
| Family communication |  |  |  |  |  |  |  |  |
| Low | 1.00 | - | 1.00 | - | 1.00 | - | 1.00 | - |
| Moderate | 1.47 | 0.74-2.95 | 1.30 | 0.59-2.86 | 1.60 | 0.68-3.75 | 1.36 | 0.52-3.54 |
| High | 2.07^*^ | 1.10-3.89 | 1.83 | 0.89-3.76 | 2.45^*^ | 1.14-5.29 | 2.61^*^ | 1.06-6.47 |

^a^Adjusted for age, income, smoking status, and depressive mood

^b^Adjusted for age, income, hypertension, diabetes, and depressive mood

^*^P < 0.05
