# Supplementary material for: Impact of family communication on self-rated health of couples who visited primary care physicians: A cross-sectional analysis of Family Cohort Study in Primary Care
Source: PLoS One. 2019 Mar 13;14(3):e0213427. doi: 10.1371/journal.pone.0213427 (PMC6415836; doi:10.1371/journal.pone.0213427)
Supplement: S3 Table — (DOCX) [file pone.0213427.s004.docx]

**S3 Table. Relationship between good SRH status and spousal combinations of family communication levels according to educational level.**

| Family communication levels (Husband + Wife) | Crude | | Multi-adjusted^a^ | |
| --- | --- | --- | --- | --- |
|  | OR | 95% CI | OR | 95% CI |
| **≤12 years of education** |  |  |  |  |
| Husbands |  |  |  |  |
| Moderate/Low + Moderate/Low | 1.00 | - | 1.00 | - |
| Moderate/Low + High | 0.83 | 0.33-2.07 | 0.82 | 0.29-2.31 |
| High + Moderate/Low | 1.21 | 0.53-2.78 | 1.10 | 0.42-2.91 |
| High + High | 1.14 | 0.57-2.26 | 0.72 | 0.31-1.68 |
| Wives |  |  |  |  |
| Moderate/Low + Moderate/Low | 1.00 | - | 1.00 | - |
| Moderate/Low + High | 1.74 | 0.78-3.89 | 2.74 | 0.90-8.35 |
| High + Moderate/Low | 0.70 | 0.31-1.60 | 1.58 | 0.52-4.81 |
| High + High | 1.57 | 0.86-2.85 | 2.51^*^ | 1.10-5.69 |
| **>12 years of education** |  |  |  |  |
| Husbands |  |  |  |  |
| Moderate/Low + Moderate/Low | 1.00 | - | 1.00 | - |
| Moderate/Low + High | 0.93 | 0.42-2.07 | 1.05 | 0.41-2.71 |
| High + Moderate/Low | 0.73 | 0.33-1.60 | 0.94 | 0.37-2.41 |
| High + High | 2.30^*^ | 1.24-4.29 | 1.86 | 0.93-3.73 |
| Wives |  |  |  |  |
| Moderate/Low + Moderate/Low | 1.00 | - | 1.00 | - |
| Moderate/Low + High | 1.19 | 0.48-2.97 | 1.77 | 0.59-5.37 |
| High + Moderate/Low | 1.03 | 0.36-2.92 | 2.05 | 0.51-8.30 |
| High + High | 2.30^*^ | 1.15-4.60 | 2.43^*^ | 1.05-5.58 |

^a^Adjusted for age, income, smoking status, hypertension, diabetes, and depressive mood

^*^P < 0.05
