# Supplementary material for: Impact of family communication on self-rated health of couples who visited primary care physicians: A cross-sectional analysis of Family Cohort Study in Primary Care
Source: PLoS One. 2019 Mar 13;14(3):e0213427. doi: 10.1371/journal.pone.0213427 (PMC6415836; doi:10.1371/journal.pone.0213427)
Supplement: S4 Table — (DOCX) [file pone.0213427.s005.docx]

**S4 Table. Relationship between family communication and health conditions.**

|  | Husbands | | | | Wives | | | |
| --- | --- | --- | --- | --- | --- | --- | --- | --- |
|  | Crude | | Multi-adjusted^a^ | | Crude | | Multi-adjusted^a^ | |
|  | OR | 95% CI | OR | 95% CI | OR | 95% CI | OR | 95% CI |
| **Diabetes Mellitus** |  |  |  |  |  |  |  |  |
| Family communication | |  |  |  |  |  |  |  |
| High | 1.00 | - | 1.00 | - | 1.00 | - | 1.00 | - |
| Moderate | 0.98 | 0.61-1.58 | 1.02 | 0.58-1.79 | 1.18 | 0.66-2.13 | 1.54 | 0.70-3.39 |
| Low | 0.60 | 0.34-1.04 | 0.57 | 0.31-1.07 | 0.39^*^ | 0.16-0.97 | 0.32 | 0.10-1.01 |
| **Hypertension** |  |  |  |  |  |  |  |  |
| Family communication | |  |  |  |  |  |  |  |
| High | 1.00 | - | 1.00 | - | 1.00 | - | 1.00 | - |
| Moderate | 1.31 | 0.84-2.04 | 1.65 | 0.96-2.82 | 0.77 | 0.48-1.21 | 0.88 | 0.47-1.66 |
| Low | 1.11 | 0.70-1.77 | 1.22 | 0.70-2.14 | 1.26 | 0.77-2.05 | 1.14 | 0.60-2.19 |
| **Dyslipidemia** |  |  |  |  |  |  |  |  |
| Family communication | |  |  |  |  |  |  |  |
| High | 1.00 | - | 1.00 | - | 1.00 | - | 1.00 | - |
| Moderate | 0.88 | 0.56-1.37 | 0.92 | 0.54-1.56 | 0.79 | 0.50-1.26 | 1.20 | 0.64-2.23 |
| Low | 0.89 | 0.55-1.43 | 0.62 | 0.35-1.10 | 0.87 | 0.53-1.45 | 1.07 | 0.56-2.04 |
| **Depressive mood** |  |  |  |  |  |  |  |  |
| Family communication | |  |  |  |  |  |  |  |
| High | 1.00 | - | 1.00 | - | 1.00 | - | 1.00 | - |
| Moderate | 2.69^*^ | 1.14-6.34 | 3.74^*^ | 1.37-10.20 | 3.10^*^ | 1.71-5.60 | 4.88^*^ | 2.11-11.27 |
| Low | 6.71^*^ | 3.05-14.77 | 7.82^*^ | 3.03-20.19 | 4.48^*^ | 2.42-8.31 | 7.83^*^ | 3.33-18.42 |

^a^Adjusted for age, educational level, income, smoking status, alcohol consumption, and physical activity

^*^P < 0.05
