# Supplementary material for: Impact of family communication on self-rated health of couples who visited primary care physicians: A cross-sectional analysis of Family Cohort Study in Primary Care
Source: PLoS One. 2019 Mar 13;14(3):e0213427. doi: 10.1371/journal.pone.0213427 (PMC6415836; doi:10.1371/journal.pone.0213427)
Supplement: S1 Appendix — (DOCX) [file pone.0213427.s006.docx]

**S1 Appendix. The Family Communication Scale of the Family Adaptation and Cohesion Evaluation Scale – IV (English version).**

1. Family members are satisfied with how we communicate with each other.

2. Family members are very good listeners.

3. Family members express affection to each other.

4. Family members are able to ask each other for what they want.

5. Family members can calmly discuss problems with each other.

6. Family members discuss their ideas and beliefs with each other.

7. When family members ask questions of each other, they get honest answers.

8. Family members try to understand each other’s feelings.

9. When angry, family members seldom say negative things about each other.

10. Family members express their true feelings to each other.

Scoring

1: Strongly disagree

2: Generally disagree

3: Undecided

4: Generally agree

5: Strongly agree
